# Ambient Listening Devices as a Feedback Tool in the Simulated Learning Environment

**DOI:** 10.7759/cureus.103222

**Published:** 2026-02-08

**Authors:** Robert Snedegar, Kendra Unger, Jason F Craig, Lauren Kozlowski, Devanie Carpenter, Emilee Pyles, Jonathan Williamson, Erika Bodkins, Dorian Williams

**Affiliations:** 1 Department of Family Medicine, West Virginia University School of Medicine, Morgantown, USA; 2 West Virginia University David and Jo Ann Shaw Center for Simulation Training and Education for Patient Safety (STEPS), West Virginia University School of Medicine, Morgantown, USA; 3 Department of Medicine, West Virginia University School of Medicine, Morgantown, USA

**Keywords:** ambient listening, artificial intelligence, formative feedback, medical education, simulation in medical education

## Abstract

Introduction: The use of artificial intelligence (AI) in medical education is expanding, yet evidence supporting its role in delivering formative feedback during simulated clinical encounters remains limited. Ambient listening (AL) technology has demonstrated utility for clinical documentation but is underexplored as an educational feedback tool. This study aimed to evaluate the feasibility, perceived quality, and learner acceptance of AI-generated feedback produced by an AL transcription system during simulated patient encounters in undergraduate medical education.

Materials and methods: First- and second-year medical students at a single U.S. medical school voluntarily participated in the study during their scheduled simulated patient encounters. Encounters were recorded using an AI-powered AL application. Students submitted a standardized skills rubric and predefined prompt to generate automated feedback and SMART goals following their encounter. Learners completed an anonymized post-encounter survey on the perceived quality of AI-provided feedback, using the validated Quality of Assessment for Learning index and optional free-text responses.

Results: A total of 10 learner evaluations were collected. All participants reported that the AI-generated feedback provided sufficient evidence of performance, included suggestions for improvement, and linked feedback directly to observed behaviors. Qualitative responses consistently described the feedback as accurate, sustainable, and closely aligned with standardized patient feedback and learner self-assessment. Suggested areas for improvement included greater contextual awareness and more specific, example-driven feedback.

Conclusions: This study is best viewed as a promising pilot study. The findings suggest AI-powered AL may serve as an easily integrated and well-accepted method for supplementing formative feedback in simulated clinical encounters. Early findings suggest it may serve as a scalable supplement to direct observation, particularly in settings with limited faculty availability. Larger, multi-institutional studies are needed to evaluate generalizability, educational impact, and effectiveness across learner levels and clinical environments.

## Introduction

The use of artificial intelligence (AI) in medical education has grown significantly in recent years. Medical students are using AI to supplement their studies, despite gaps in our knowledge of AI accuracy and limited understanding of its educational outcomes in the clinical learning environment [1]. Furthermore, many leading review articles call for further research on how to integrate AI into a standardized framework within medical education while also educating learners and educators about the biases inherent in AI results [2-5]. The current literature suggests that AI can provide personalized and timely feedback, thereby improving students’ writing skills and overall performance in both clinical and simulated learning environments [6-10].

Direct observation (DO) is an educational technique frequently used in medical education. A recent study confirms that DO is necessary to bridge the gap between students' knowledge and its application in clinical settings [11]. Despite its advantages, the use of DO is often limited to formal assessment because learners perceive a loss of autonomy during observation and because such assessments require substantial time. Additionally, other studies suggest the fast-paced demands of the clinical environment limit the use of DO and demonstrate that students prefer a written formative feedback approach [12-13].

Ambient listening (AL) is a relatively new technology that harnesses the capabilities of electronic devices equipped with microphones to capture conversations between patients and medical professionals and to document them thoroughly and succinctly. In recent years, substantial research on the accuracy of AL in clinical settings and its utility for replacing medical scribes has been published [14-16]. Interestingly, a recent study demonstrated that wearable sensors can improve patient-clinician communication in a scripted simulated learning environment [17]. Despite this, AL’s utility in providing feedback for learners remains underexplored. To date, no studies indexed in PubMed have described student evaluations of AI feedback quality in simulated learning environments for undergraduate medical education.

A previous study showed that GPT-4, a large language model, could “simulate patient interactions and provide tailored, unsupervised feedback to medical students”. It showed that there was “almost perfect” agreement between the feedback provided by an AI and that of a human rater [9]. This project was designed to investigate the integration of an AI-powered AL transcription tool as a feedback generator within a simulated clinical learning environment and to evaluate medical students' attitudes toward this feedback technology with respect to technical usability and student acceptance.

## Materials and methods

First- and second-year medical students at a single U.S. medical school were recruited to the study via a mass email sent to the class-wide mailing list. Students were invited to voluntarily participate in the study, which supplemented regularly scheduled simulated patient encounters in their clinical skills course with feedback from an AL device. Students did not receive compensation for their participation in the study. Their grades and class standing were not affected by participation or non-participation. Interested students contacted the study authors via email and completed a one-hour training session on the software. During the training, students viewed a simulated patient encounter recorded by the AL technology Otter (Otter.ai, Inc., Mountain View, CA, USA). They were then instructed to enter the prewritten prompt into the AL device’s chatbot and to review the chatbot's feedback. Following the training, 10 students volunteered to participate in the study. Students provided written consent, created an Otter account, and downloaded the application to their personal mobile devices. All standardized patients (SPs) with whom participating students were scheduled to have a simulated encounter consented in writing to being recorded with the AL device. The West Virginia University Institutional Review Board issued approval 2308828386.

Students then participated in simulated history and physical examinations in the University’s Center for Simulation Training and Education for Patient Safety (STEPS) as part of their clinical skills course. Upon entering STEPS, students participating in the study used the AL application on their mobile devices to record the simulated patient encounter and completed it as usual. To maintain compliance with the Family Educational Rights and Privacy Act (FERPA) and the Health Insurance Portability and Accountability Act (HIPAA), students were asked not to use their full names during the encounter, and SPs used pseudonyms. Upon returning home, students logged into their AL account on a desktop or laptop, placed the uniform, predetermined standardized feedback rubric for the respective clinical skills course SP encounter in the AL interface, and submitted the following prompt to the chatbot: “Please provide me with quality constructive feedback as the provider in this encounter. Additionally, please provide me with three SMART goals for improvement.” The chatbot then provided each student with instantaneous feedback.

Finally, students completed a four-question anonymized survey on the feedback they received from AL. The survey consisted of the validated QuAL index tool as well as a free-text box with the question, “Please use this space to describe how you feel about receiving feedback from Otter.AI (i.e., quality, comfort, sustainability)” [18]. Questions from the validated QuAL index tool included, “Does the rater provide sufficient evidence about performance?” (scored as yes = three points, maybe = two points, no = one point, no comment = zero points): “Does the rater provide a suggestion for improvement?” (scored as yes = one point, no = zero points); and “Is the rater’s suggestion linked to the behavior described?” (scored as yes = one point, no = zero points).

## Results

Study results were collected at the end of all SP encounters for students who volunteered to participate. A total of 10 learner evaluations were collected. Across all 10 encounters, every learner indicated that the AI agent provided sufficient evidence about performance, offered at least one suggestion for improvement, and that the suggestion was clearly linked to the behavior described (Table 1). Qualitative comments from evaluations reflected consistent themes, including descriptions of the feedback as sustainable, accurate, and direct (Table 2). Students also provided feedback for improvement, including the use of more context-rich prompts for the AL chatbot. Because all participants provided identical maximum scores, variability-based visualizations were not appropriate; the results are presented descriptively.

**Table 1:** QuAL index scores QuAL: Quality of Assessment for Learning, SD: standard deviation, IQR: interquartile range

Question	Measure	n	Mean (SD)	Median (IQR)
Does the rater provide sufficient evidence about performance?	QuAL index score (3 = yes, 2 = maybe, 1 = no, 0 = no response)	10	3 (0.0)	3 (3-3)
Does the rater provide a suggestion for improvement?	QuAL index score (1 = yes, 0 = no)	10	1 (0.0)	1 (1-1)
Is the rater’s suggestion linked to the behavior described?	QuAL index score (1 = yes, 0 = no)	10	1 (0.0)	1 (1-1)
Total aggregate index score	QuAL index scores	10	5 (0.0)	5 (5-5)

**Table 2:** Student's free text responses to AL feedback AL: ambient listening, SP: standardized patient, AI: artificial intelligence

Student free text response
	“It was quality feedback, but it was what my SP told me as well. That is helpful to know that my SP gave feedback that a standardized AI system would also. The feedback is definitely sustainable and will help me to improve for next time.”
“I think Otter AI provided good feedback, and suggestions for improvement aligned with my own self-assessment of this experience.”	
	“The quality of my feedback was very helpful. I can see improvement from how my last one went and can see where I should further improve. The ease of use is nice, however I forgot to stop my recording so some of my feedback from the SP was on the transcript and guided the AI response. I wish there was a way to delete some of the transcript to exclude in the feedback.”
“I was very impressed with Otter AI features because the feedback it gave me was almost identical to that from my SP. All of the information provided was very concise and the SMART goals were perfectly laid out for what exact steps I can take next time to take an even better history. “	
	“The feedback was very direct and attainable. I feel I can implement these practices and see markable improvements.”
“I like the feedback from Otter AI, it's helpful constructive criticism mixed in with telling me what I did well. I think it might be more helpful if Otter AI was informed what the purpose of today's exercise was so that it gives appropriate feedback. For example, it said I should've mentioned what the patient's next steps should be/treatment plan but today was just a practice history. otherwise, helpful.”	
	“I think the feedback Otter AI provides is very helpful. With the SMART goals I feel like I know exactly what I can improve, which is a relief because the actor gave me minimal feedback. I would keep using this tool in my patient encounters and would recommend it to other colleagues. I would like to see Otter AI have more explanations in its feedback of specific examples of things I can do to improve. Right now it is a little vague with more concepts than actual examples. I think with a few more prompts in the AI Chat it would be easy to get this more detailed feedback.”
“I felt that the feedback was very accurate to things I discussed with my SP and also things I determined after self-review of the video recording. Using the AI feature was very easy and simple to navigate. It gave instant feedback that I could immediately begin using to formulate a plan to do even better for the next time. Overall, the quality was great as it reflected not only things that can be picked up through standard audio but even mentioned .”	

In this early implementation, learners consistently endorsed AI feedback as accurate, behaviorally linked, and improvement-oriented, with universal agreement that it provided sufficient evidence and actionable suggestions. Our study outlines the effectiveness, feasibility, and student acceptance of AI-powered AL as an alternative feedback mechanism in medical education.

## Discussion

This study expands the emerging literature on AI in medical education by examining the novel use of AL technology as a formative feedback tool in simulated clinical encounters. While prior research has demonstrated the utility of AI for documentation, assessment support, and simulated patient interactions, few studies have explored its role in delivering structured, behavior-linked feedback to undergraduate medical students during skills training [6-10]. Our findings suggest that AL-generated feedback is not only feasible and well accepted by learners but also aligns closely with established principles of high-quality formative feedback.

Consistent with prior literature, participants in this study perceived AI-generated feedback as accurate, actionable, and linked to observable behaviors. Key characteristics of effective feedback are outlined in educational theory and validated assessment frameworks such as the QuAL score. Chan et al. demonstrated that feedback is most impactful when it provides sufficient evidence of performance and clear suggestions for improvement, both of which were universally endorsed by learners in our cohort [18]. These findings parallel earlier studies showing that AI-generated feedback can support reflective learning and skill development when appropriately structured and aligned with educational objectives [6-10].

Our results also align with prior investigations of large language models in simulated medical education. Holderried et al. demonstrated that a language-model-powered simulated patient could deliver automated feedback on history-taking skills, with high learner acceptance and educational plausibility [9]. Similarly, earlier work using GPT-4 demonstrated near-perfect agreement between AI-generated feedback and human raters in simulated patient encounters, supporting the credibility of AI as an adjunct to human feedback [7]. Learners in our study echoed these findings, frequently noting close alignment between AL-generated feedback, SP comments, and their own self-assessments.

Notably, the use of AL as a feedback mechanism may address longstanding limitations associated with DO in medical education. Although DO is widely regarded as essential for bridging the gap between knowledge and clinical performance, its implementation is often constrained by faculty time, learner discomfort, and clinical workflow pressures. Prior studies have shown that learners frequently prefer written formative feedback and that sociocultural factors can limit the effectiveness and frequency of DO in practice [11-13]. Our findings suggest that AL-generated feedback may supplement DO, providing timely, individualized written feedback without increasing faculty workload.

The positive learner perceptions observed in this study are consistent with broader literature examining student attitudes toward AI in medical education. Recent surveys indicate that medical students generally view AI as credible and effective when used transparently and in support of learning rather than summative evaluation [8]. Students in our cohort similarly emphasized the sustainability, ease of use, and immediacy of AL feedback, characteristics associated with improved learner engagement and self-directed improvement in prior studies of AI-supported feedback on written and clinical tasks.

Despite these promising findings, learners identified limitations that mirror concerns raised in prior research on AI education. Specifically, students noted a lack of contextual awareness and a desire for more specific, example-driven feedback. Similar challenges have been reported in studies of AI-generated educational feedback, underscoring the importance of carefully designed prompts, explicit learning objectives, and human oversight to ensure contextual relevance. These findings suggest that AI feedback tools are most effective when integrated thoughtfully into curricular frameworks and used as complements rather than replacements to human instruction and coaching.

Notably, this study has several limitations. The sample size is very small, with only 10 encounters, due to low voluntary participation among the study population, resulting in a low-powered study. All encounters involve medical students in the first- and second-year didactic phase of their training and may not be generalizable to more advanced learners. Additionally, this is a single-institution study, which may limit generalizability to other institutions’ learning models. Over-reliance on the proprietary AI platform and the institution-specific simulation workflows of the study institution may limit the reproducibility of results when applied in other settings. All students rated the feedback with a perfect score, which may indicate acquiescence bias, as students knew how the AL device's feedback was measured. Finally, the model was tested only in SP encounters within the STEPS simulation center. This limits conclusions regarding the tool’s applicability across diverse settings, including the live clinical environment.

## Conclusions

This is the first study to explore the technical usability and student acceptance of AL feedback in the simulated learning environment. Overall, student feedback was positive, demonstrating AL's strong potential to complement medical education by providing credible, effective feedback to improve students' clinical and communication skills during practice encounters with SPs. Moreover, such tools can help scale formative feedback for medical trainees when faculty time is limited. Such feedback is essential for the early development of reflective practice, communication skills, and confidence, thereby preparing students for patient-centered care during clinical clerkships and beyond. Nonetheless, given the very small sample size, this study should be viewed as a promising pilot that must be replicated with a larger number of learners across multiple institutions to strengthen external validity.

Future studies should focus on recruiting more learners to participate, including those in clerkship phases of their medical training, and testing the model’s efficacy in other contexts. However, this remains limited by the current HIPAA-compliant AL devices’ ability to provide high-quality feedback on patient encounters. Additionally, clinicians should continue to investigate students' perceptions of the integration of AI and AL into their medical education and assess the quality of feedback provided in a blinded manner to prevent acquiescence bias that may have affected the results of this study. A particularly interesting study topic would be to compare student ratings of faculty feedback with AL feedback. Longitudinal studies comparing the performance of students who are exposed to AI and AL devices early in training with those who are not may also prove interesting as these technologies continue to become more integrated into the medical field.
